# Preadmission assessment of extended length of hospital stay with RFECV-ETC and hospital-specific data

**DOI:** 10.1186/s40001-022-00754-4

**Published:** 2022-07-25

**Authors:** Chinedu I. Ossai, David Rankin, Nilmini Wickramasinghe

**Affiliations:** 1 School of Health Sciences, Department of Health and Biostatistics, Swinburne University, John Street Hawthorn, Victoria, 3122 Australia; 2 Cabrini Health, Melbourne, Australia

**Keywords:** Extended length of hospital stay, Recursive feature elimination, Extra tree classifier, Multivariate logistic regression, Admission risk

## Abstract

**Background:**

Patients who exceed their expected length of stay in the hospital come at a cost to stakeholders in the healthcare sector as bed spaces are limited for new patients, nosocomial infections increase and the outcome for many patients is hampered due to multimorbidity after hospitalization.

**Objectives:**

This paper develops a technique for predicting Extended Length of Hospital Stay (ELOHS) at preadmission and their risk factors using hospital data.

**Methods:**

A total of 91,468 records of patient’s hospital information from a private acute teaching hospital were used for developing a machine learning algorithm relaying on Recursive Feature Elimination with Cross-Validation and Extra Tree Classifier (RFECV-ETC). The study implemented Synthetic Minority Oversampling Technique (SMOTE) and tenfold cross-validation to determine the optimal features for predicting ELOHS while relying on multivariate Logistic Regression (LR) for computing the risk factors and the Relative Risk (RR) of ELOHS at a 95% confidence level.

**Results:**

An estimated 11.54% of the patients have ELOHS, which increases with patient age as patients < 18 years, 18–40 years, 40–65 years and ≥ 65 years, respectively, have 2.57%, 4.33%, 8.1%, and 15.18% ELOHS rates. The RFECV-ETC algorithm predicted preadmission ELOHS to an accuracy of 89.3%. Age is a predominant risk factors of ELOHS with patients who are > 90 years—PAG (> 90) {RR: 1.85 (1.34–2.56), *P*:  < 0.001} having 6.23% and 23.3%, respectively, higher likelihood of ELOHS than patient 80–90 years old—PAG (80–90) {RR: 1.74 (1.34–2.38), *P*:  < 0.001} and those 70–80 years old—PAG (70–80) {RR: 1.5 (1.1–2.05), *P*: 0.011}. Those from admission category—ADC (US1) {RR: 3.64 (3.09–4.28, *P*:  < 0.001} are 14.8% and 70.5%, respectively, more prone to ELOHS compared to ADC (UC1) {RR: 3.17 (2.82–3.55), *P*:  < 0.001} and ADC (EMG) {RR: 2.11 (1.93–2.31), *P*:  < 0.001}. Patients from SES (low) {RR: 1.45 (1.24–1.71), *P*:  < 0.001)} are 13.3% and 45% more susceptible to those from SES (middle) and SES (high). Admission type (ADT) such as AS2, M2, NEWS, S2 and others {RR: 1.37–2.77 (1.25–6.19), *P*:  < 0.001} also have a high likelihood of contributing to ELOHS while the distance to hospital (DTH) {RR: 0.64–0.75 (0.56–0.82), *P*:  < 0.001}, Charlson Score (CCI) {RR: 0.31–0.68 (0.22–0.99), *P*:  < 0.001–0.043} and some VMO specialties {RR: 0.08–0.69 (0.03–0.98), *P*:  < 0.001–0.035} have limited influence on ELOHS.

**Conclusions:**

Relying on the preadmission assessment of ELOHS helps identify those patients who are susceptible to exceeding their expected length of stay on admission, thus, making it possible to improve patients’ management and outcomes.

## Introduction

When a patient stays more than three times the average length of stay (LOS) for a given Diagnosis Related Group (DRG), the patients will be said to have an Extended Length of Hospital Stay (ELOHS). Thus, making it imperative that patients are managed effectively in the hospitals to prevent them from exceeding their expected length of stay since that will introduce more financial burdens on the hospitals, health insurance, and the government [6, 23, 24] as well as causes more health complications for patients due to nosocomial infections [22]. There is a widespread variation in patients length of stay (LOS) in many public hospitals due to some inefficiencies associated with understanding and managing patients effectively from admission to discharge. This has resulted in significant cost blowout due to lack of hospital bed spaces that resulted in the loss of $125 M per annum to service patients overstaying on admission in the State of Victoria Australia [25]. This information makes it imperative that hospitals seek ways of reducing ELOHS through better knowledge of patients’ clinical and psychosocial features that may lead to the identification of high-risk patients and make it easier to provide appropriate care.

There are numerous studies on ELOHS, and their associated risk factors. Burton et al. [21] predicted ELOHS for patients of percutaneous coronary intervention using multivariate Logistic Regression (LR) by taking Normal Length of Hospital Stay (NLOHS) as patients staying < 5 days and those staying ≥ 5 days as ELOHS and obtained a prediction accuracy (AUC) of 79.9–81.9%. Staziak et al. [16] obtained an accuracy of 80—81% for ELOHS prediction of torso trauma patients using clinical and image data with Support Vector Machine (SVM) and Artificial Neural Network (ANN) algorithms. Zhang et al. [20] predicted the prospects of ELOHS for adult spinal deformity patients undergoing posterior spinal fusion surgery to an accuracy of 68–83% using LR, Decision Tree classifier (DTC), Random Forest (RF), XGBoost (XGB), and Gradient Boosting Machine (GBM) by considering ELOHS as those spending > 9 days in the hospital. Zolbanin et al. [26] predicted the length of stay (LOS) for patients suffering from chronic obstructive pulmonary disease (COPD) and pneumonia with a deep neural network algorithm and obtained an accuracy of 86–91% for COPD and 74–85% for pneumonia.

Numerous studies on ELOHS have described ELOHS as a specific number of days in the hospital that corresponds to the 75th percentile of the studied cohorts [9, 16, 21] while others chose a particular number of days in the hospital as the limit for NLOHS for a combination of DRGs [30, 31, 35]. Unfortunately, the variability in the severity of health conditions with various DRGs makes it imperative to consider ELOHS as a DRG-specific definition requiring specific durations. This approach is used in this study to define ELOHS for the various DRGs considered.

Even though better management of patients can be crafted from numerous conditions that include the DRG, patients’ demographic and clinical information, and several psychosocial conditions [6–9], patients’ susceptibility to ELOHS must be known preadmission if they are to be better managed. This will allow the hospitals to develop requisite patients’ management plans ab initio and forestall using ineffective strategies that may lead to ELOHS. Unfortunately, numerous ELOHS and risk factors prediction models did not consider hospital-specific factors and were not designed for preadmission. To this end, this study aims to utilize hospital-specific clinical and demographic features and documented psychosocial attributes of the patients to develop a machine learning technique for the ready prediction of ELOHS. The risk factors for ELOHS were determined based on the considered features to facilitate better patient management. The study relied on Recursive Feature Elimination with Cross-Validation and Extra Tree Classifier (RFECV-ETC) to predict ELOHS while using multivariate LR for estimating the risk factors and relative risk (RR) of ELOHS at a 95% significant level.

The fact that ELOHS is linked to numerous unpleasant outcomes in hospitalization such as decreased survival rate, increased time in the intensive care unit (ICU), increased number of hospital visits, preadmission comorbidities, infections, and complications [6, 8, 9, 33, 34] makes it imperative to understand patients’ ELOHS susceptibility preadmission. To this end, the strategy developed in this study will go a long way to promote improved patients experience seeing that the chances of developing and implementing contingency plans for patients’ care to forestall prolonged hospital stay will be executed at admission. The contribution of this study can be summarized as:

- Design and development of a predictive technique for understanding patients’ susceptibility to ELOHS preadmission, which allows for the implementation of best practices in patient care to forestall extended hospital stay.
- Using a definitive description of ELOHS to identify patients on admission who may be at risk of extended stay rather than adopting a specific LOS as the boundary between ELOHS and normal LOS as exemplified by numerous researchers [9, 16, 20, 35].
- The use of Recursive Feature Elimination with Cross-Validation and Extra Tree Classifier (RFECV-ETC), to help determine the optimal features that will contribute to ELOHS prediction without overfitting the model.
- Provision of risk factors and relative risk categorizes of the hospital and patient’s demographic and psychosocial characteristics enables reflective practice on patients’ management that will be vital for reducing hospital-acquired complications and infections.
- This study provides a better outlook for ELOHS by developing a strategy for understanding the proneness of all patients suffering from different DRGs to ELOHS on admission.

## Methodology

The ELOHS used in this study is defined as 3* average length of stay (ALOS) for a given DRG following the Independent Hospital Pricing Authority (IHPA) standard national pricing model [39]. This specification is based on the resource use, diagnosis, and procedure coding for different DRGs and follows the “L3H3” trimming method and modifications that account for different adjustments according to episode severity. The advantage of defining the ELOHS with the “L3H3” is the ability to clearly describe a billing framework that equitably accounts for DRGs across hospitals nationally, thus, creating room for clinical cost normalization across the hospitals. Imperatively, patients, hospitals, and insurance companies are treated equitably when it comes to the cost of managing patients treated for different episodes.

This study develops a machine learning model for predicting patients prone to ELOHS preadmission using hospital, demographic, and psychosocial features. To ensure proper reporting that follows the prescribed benchmarks for modelling projects in medical informatics, the technique developed by Cabitza and Campagner [38] was adopted for self-assessment of the work. The breakdown of the procedure used for acquiring and pre-processing the data used for the study, the modelling strategy, and the statistical analysis approach for determining the risk factors of ELOHS are shown in the following sub-sections.

### Data acquisition

De-identified patients’ records were obtained for separations between 10/2015 and 12/2020 from a private acute teaching hospital in Australia. Data were sourced from the Hospital Casemix Protocol (HCP) data extract routinely supplied to regulators from the patient administration data set. Initially, 91,468 samples comprising 73 features were extracted from the data set before the pre-processing that eliminates the features that have no relevance to the preadmission assessment of patients’ ELOHS. This process helped narrow down the features to the following hospital-specific parameters—visiting medical officer (VMO) specialty, patient age, patient gender, admission category (ADC), admission type, patient care class, Charlson Score, socioeconomic status (SES), and distance to hospital (DTH).

### Data processing

Patient records with missing values were dropped from the dataset to minimize the impact of replacing missing values on the model accuracy. The features were also categorized into subclasses while the postcodes are used for computing the SES and DTH. The Socio-Economic Indexes for Areas (SEIFA) from the Australian Bureau of Statistics (ABS) [28] are used for classifying the patients as low (1–4 decile), middle (5–7 decile), and high (8–10 decile) SES. The longitude and latitude of the hospital and the patient's postcode provided the information for calculating DTH using the great circle distance model of the earth [29] for the Global Positioning System (GPS) location. To reduce the influence of outliers and extreme values in the model, VMO specialties with less than 100 samples were merged as a new VMO specialty class called VMO-others. The same process was used for admission types and patients religion by combining the classes with less than 100 samples to create new classes of the features. For Charlson Score, those with a score of 8 and above were merged to create a unique subclass (> 8). However, for admission category, the classes with less than 200 samples were merged to produce a new class. Since all independent features are categorical parameters, the various samples are represented as “1” for a given feature when the sample is affirmative for such feature subclass of “0” if the condition is negative. For instance, for the Patient Age subclass > 90 years, a sample row with a patient of age 45 years will have “0” while the row with patient age of 91 years will have “1”. The 10 features and their corresponding number of subclasses that are used in this study to model ELOHS are shown in Table 1.

**Table 1:** Modelling features and their number of classes after pre-processing

Features	Acronym	Number of classes
VMO specialty	VMO	33
Patient age	PAG	9
Patient gender	PGD	2
Admission category	ADC	6
Admission type	ADT	15
Patient care class	PCC	3
Patient religion	PRG	15
Distance to hospital	DTH	4
Socioeconomic status	SES	3
Charlson Score	CCI	9

### *Prediction of preadmission ELOHS*

To predict the ELOHS of patients' preadmission involves determining the likelihood of a patient exceeding their expected stay by considering the hospital-specific and psychosocial features itemized in Table 1. This is done by training and testing a machine learning model using the pre-processed data. The first step is to balance the data using the Synthetic Minority Oversampling Technique (SMOTE) [27] that ensures that the target feature (ELOHS) is of the same size amongst those with extended stay (‘1’) and those whose stay was within expected limits (‘0’). SMOTE has the potentials of reducing the class imbalance problems that include poor true positive and negative rates estimation, and model underfitting because of the poor learning performance of algorithms [1, 2]. As soon as the imbalance problem is solved, the next step is to determine the best model to use in the modelling of ELOHS by considering all the features shown in Table 1. Please note that the 99 sub-features of all the features are considered at this stage. Some of the prominent algorithms used for solving health informatics problems relating to ELOHS and LOS are tried on the dataset by implementing tenfold cross-validation. Implementing cross-validation ensures that all the sections of the dataset are used for training and testing the model. This practice makes it possible to have a better picture of the model’s performance because the accuracies of all the fields considered in the training and testing exercise are considered in computing the accuracy of the model. The best performing algorithm is determined by comparing the accuracy measured as sensitivity, specificity, and F1-score. The best algorithm is later used as the base estimator Recursive Feature Elimination with Cross-Validation (RFECV) in a stepwise fashion that considered different combinations of the features shown in Table 1. The algorithms tried at this stage include Extra tree Classifier (ETC), XGBoost (XGB), Adaptive Booster (ADB), GBM, ANN, RF, Support Vector Machine (SVM), and DTC. Since the best algorithm for predicting the accuracy of ELOHS amongst the tried algorithms is ETC, this study relied on ETC as the base estimators for the RFECV. The next section shows the strategy for obtaining the optimal features for ELOHS prediction using the RFECV-ETC model.

### *Optimal features selection (OFS) with RFECV-*ETC

The RFECV is a backward elimination method that starts with a full set of all features and then removes the most irrelevant features one by one based on the validation scores [3]. This process aims to get the optimal number of features that will result in the best model accuracy by eliminating the features that have not influenced the accuracy. The procedure for using RFECV-ETC for predicting the best features combinations and the sub-features to obtain optimal accuracy of ELOHS is shown in Algorithm 1.
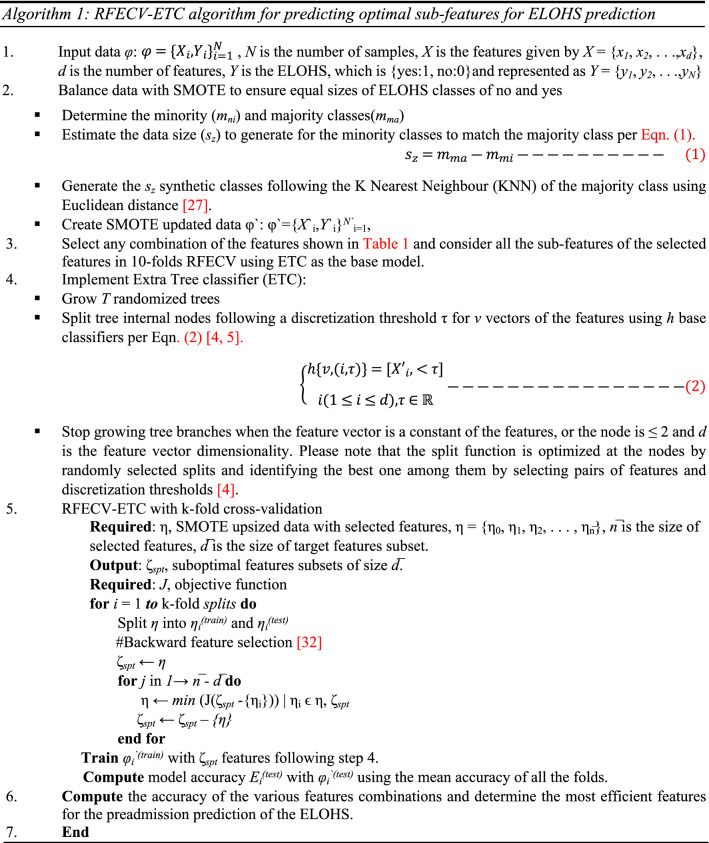


### Risk factors estimation

The ELOHS risk factors are identified as a way of allowing the doctors and other health experts to know the high-risk patients who need specific attention because of their likelihood of extended stay on admission. Multivariate LR is used for computing the relative risk (RR) of ELOHS at a 95% confidence level with features having P-value ≤ 0.05 considered as significant risk factors. The appropriateness of the multivariate LR is assessed using accuracy computation that considered the true-positive, false-positive, true-negative, and false-negative predictions of the model for all the data, training data (70%), and testing data (30%). The computation of the risk factors and RR is based on the 10 features considered in the study.

## Results

### Descriptive statistics of features

A total of 33,752 records are used from the initial 91,468 records after the pre-processing. There are 11.54% of ELOHS patients, which increased with the age of the patients. For instance, patients who are ≥ 65 years are 87%, 251%, and 491%, respectively, more prone to ELOHS than those < 18 years, 18–40 years, and 40–65 years. Female patients have a slightly higher (2.12%) ELOHS rate than males (10.33%) while patients with Charlson score of 5–8 have a higher ELOHS rate than others. The higher the length of stay (LOS) the higher the rate of ELOHS. This is evidence in the rate of ELOHS for health conditions that warranted > 20 days LOS with 71.15% ELOHS rate compared with other patients with ≤ 5 LOS (ELOHS rate: 0.41%), 6–10 days LOS (ELOHS rate: 19.69%), and 11–20 days LOS (ELOHS rate: 47.28%). A summary of some of the features used in this study and the frequencies of the NLOHS and ELOHS are shown in Table 2.

**Table 2:** Summary of frequencies (%) of some of the features considered in the model

Features	NLOHS (%)	ELOHS (%)
Total population	29,856 (88.46%)	3896 (11.54%)
*Patient age*		
Under_18	1481 (97.43%)	39 (2.57%)
18–40	4127 (95.67%)	187 (4.33%)
40–65	7354 (91.9%)	648 (8.1%)
65 and over	16,893 (84.82%)	3023 (15.18%)
*Patient gender*		
Female	16,912 (87.55%)	2406 (12.45%)
Male	12,943 (89.67%)	1491 (10.33%)
*Length of stay (LOS)*		
≤ 5 days	23,428 (99.59%)	96 (0.41%)
6-10 days	4288 (80.31%)	1051 (19.69%)
11–20 days	1609 (52.72%)	1443 (47.28%)
> 20 days	530 (28.85%)	1307 (71.15%)
*Charlson Score*		
0–1	10,224 (94.05%)	647 (5.95%)
2–4	14,987 (87.46%)	2149 (12.54%)
5–8	3948 (80.67%)	946 (19.33%)
> 8	696 (81.79%)	155 (18.21%)
*VMO (visiting medical officer) specialty*		
Cardiology	2870 (88.8%)	362 (11.2%)
Colorectal surgery	1434 (87.65%)	202 (12.35%)
Endocrinology	116 (53.21%)	102 (46.79%)
Gastroenterology	1960 (85.07%)	344 (14.93%)
Gynaecology	788 (92.49%)	64 (7.51%)
Haematology	258 (65.82%)	134 (34.18%)
Medical oncology	502 (69.53%)	220 (30.47%)
Nephrology	274 (45.67%)	326 (54.33%)
Neurology	166 (51.88%)	154 (48.13%)
Neurosurgery	2170 (95.51%)	102 (4.49%)
Obstetrics & Gynae	1876 (98.01%)	38 (1.99%)
Orthopaedic surgery	5776 (90.59%)	600 (9.41%)
*Distance to hospital (DTH)*		
> 20 km	6353 (89.42%)	752 (10.58%)
5-10 km	7688 (88.36%)	1013 (11.64%)
0-5 km	9907 (88.16%)	1330 (11.84%)
10-20 km	5907 (88.05%)	802 (11.95%)
*Socioeconomic status (SES)*		
High	11,416 (77.73%)	3270 (22.27%)
Middle	2607 (88.92%)	325 (11.08%)
Low	2115 (87.58%)	300 (12.42%)

### Prediction of ELOHS with RFECV-ETC

RFECV is a feature selection technique that uses a recursive process for ranking features according to their importance and uses elimination to exclude weak features, dependencies, and collinearities from a model to improve the prediction accuracy [40]. By the process of cross-validation, the optimal features for enhanced performance are identified after dropping the insignificant features that are not positively impacting the model accuracy. As stated previously, eight algorithms that include GBM, ETC, RF, XGB, ANN, DTC, ADB, and SVM are considered in this study in the first instance to identify the one that will be most appropriate for predicting ELOHS. The result of the tenfold cross-validation of SMOTE data measured with recall, precision, and F1-score is shown in Table 3 (see Appendix 1 for the characteristics of the various algorithms). Since ETC is the best performing algorithm, future analysis to determine the optimal features for predicting ELOHS is done with ETC as the base algorithm for RFECV.

**Table 3:** Comparison of prediction accuracy of ELOHS using tenfold cross-validation for ELOHS with all the features

Algorithm	Recall	Precision	F1-score	Balanced accuracy	ROC AUC
KNN	0.807 ± 0.045	0.821 ± 0.032	0.803 ± 0.05	0.807 ± 0.045	0.892 ± 0.033
GBM	0.746 ± 0.12	0.77 ± 0.1	0.732 ± 0.136	0.746 ± 0.12	0.876 ± 0.075
DTC	0.818 ± 0.061	0.845 ± 0.054	0.814 ± 0.063	0.818 ± 0.061	0.907 ± 0.054
ADB	0.715 ± 0.104	0.732 ± 0.09	0.703 ± 0.118	0.715 ± 0.105	0.835 ± 0.078
**ETC**	**0.885 ± 0.063**	**0.9 ± 0.052**	**0.883 ± 0.066**	**0.885 ± 0.063**	**0.952 ± 0.039**
SVM	0.723 ± 0.076	0.726 ± 0.072	0.719 ± 0.083	0.723 ± 0.076	0.805 ± 0.07
XGB	0.77 ± 0.11	0.809 ± 0.083	0.755 ± 0.128	0.77 ± 0.11	0.927 ± 0.075
RF	0.859 ± 0.078	0.883 ± 0.061	0.855 ± 0.082	0.859 ± 0.078	0.953 ± 0.053

**Fig. 1:**
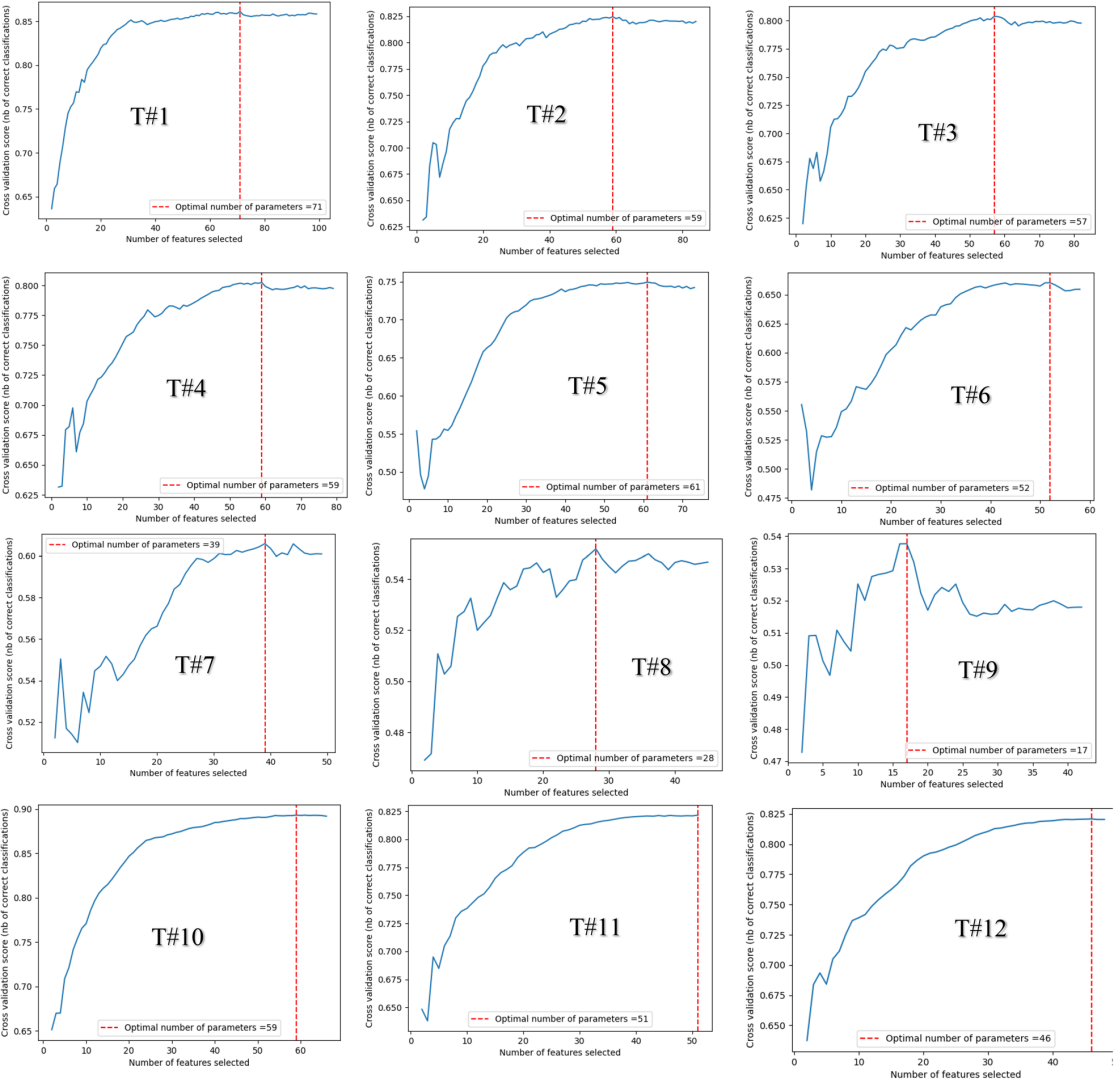
The mean performance scores and optimal features selection points for tenfold cross-validation of the RFECV-ETC algorithm for the numerous combinations of the features

Since there is a likelihood of improving ELOHS prediction accuracy by relying only on the features and sub-features that have enhanced correlation with the target feature, the stepwise comparison of the features using their sub-features in the RFECV-ETC algorithm is shown in Table 3 (the mean performance scores and the point of optimal features selection for the tenfold cross-validation of the trials can be viewed in Fig. 1).

The features considered in the optimal performance modelling are marked (√), whereas those that are not considered are marked (x). Each of the trials produced the optimal number of features to better ELOHS prediction. The modelling attempt (*T#10*), which considered the following input features PAG, PGD, ADC, ADT, PCC, PRG, DTC, SES and CCI produced the best accuracy of 89.3%. This prediction accuracy is comparably higher than some of the prediction models for ELOHS carried out previously as shown in the following references [16, 20, 21].

Although the optimal feature selection points shown in Fig. 1 represent the optimal number of features that will guarantee the best prediction of ELOHS for the 10 features (99 sub-features) or less with a stepwise combination of the features (as shown in Table 3), there may be the need for a trade-off when the accuracy at the optimal features point increases minimally. For instance, in T#1, when 31 sub-features are used (Fig. 1- T#1), the accuracy was 85.34% and when the optimal solution was found at 71 sub-features, the accuracy is 86%, which is an increase of 0.66%. Since the inclusion of additional 40 parameters in the algorithm training will increase the computational cost, it may suffice to trade-off the 0.66% extra accuracy for fewer parameters especially when the size of the data increases disproportionately as expected when the algorithm is deployed into production.

**Fig. 2:**
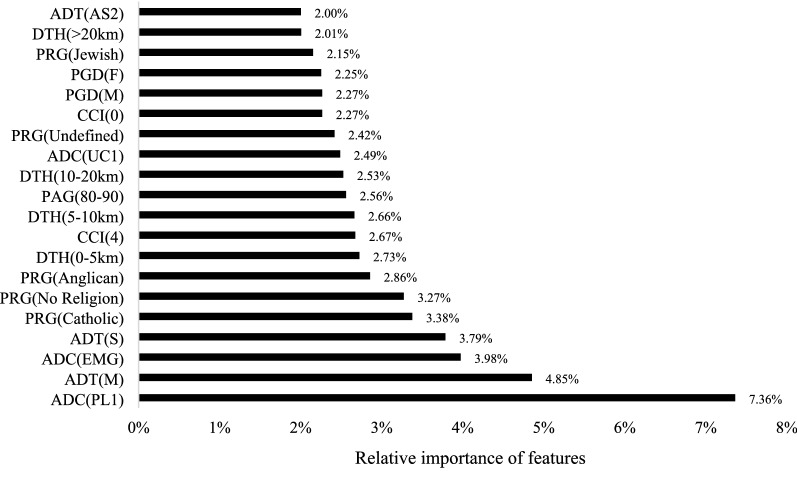
Summary of the 20 best features for the best predictive models (T#10)

The first 20 most important features of the best-performing trial model (*T#10*) are shown in Fig. 2. With a 2.00–7.36% relative importance of these features, they have 0.07–5.85 times more importance than the 21–40^th^ important features of the model and 1.92–31.41 times better than the 41–59th most important features. Even though the less important features did not contribute much to the accuracy of the model, excluding them will reduce the prediction accuracy of ELOHS, and adding other features that are not part of the selected optimal features will also reduce the prediction accuracy.

Since the RFECV-ETC algorithm relies on tenfold cross-validation to predict patients’ ELOHS status, it suffices to show the performance accuracies of the target feature of the best performing model (T#10) as another way of ascertaining the model’s performance for the ELOHS and NLOHS patients. Table 4 summarizes the performance of the various folds in the cross-validation and how good ELOHS and NLOHS are predicted with RFCV-ETC. With an accuracy of 76.12–94.52% across the folds for all the target feature (NLOHS and ELOHS) and almost even accuracy levels for NLOHS and ELOHS for each fold, the RFCV-ETC can be acclaimed to have properly modelled ELOHS since there is no substantial lapse in prediction accuracy at any section of the dataset.

**Table 4:** Summary of the recall, precision, accuracy, and the optimal features selection (OFS) obtained with RFECV of the various input features combinations represented as a trial number (T#), √: included, x; exclude, ACC: accuracy, BACC: balanced accuracy, AUC: area under the curve, RCL: recall, PRC: precision

Features	T#1	T#2	T#3	T#4	T#5	T#6	T#7	T#8	T#9	T#10	T#11	T#12
VMO	√	√	√	√	√	√	√	√	√	x	x	x
PAG	√	√	√	√	√	√	√	√	√	√	√	√
PGD	√	√	x	x	x	x	x	x	x	√	√	√
ADC	√	√	√	√	x	x	x	x	x	√	√	√
ADT	√	√	√	√	√	x	x	x	x	√	√	√
PCC	√	√	√	x	x	x	x	x	x	√	√	x
PRG	√	** × **	** × **	** × **	** × **	** × **	** × **	** × **	** × **	√	x	x
DTH	√	√	√	√	√	√	√	** × **	** × **	√	√	√
SES	√	√	√	√	√	√	√	√	** × **	√	√	√
CCI	√	√	√	√	√	√	** × **	** × **	** × **	√	√	√
OFS	71	59	57	59	61	52	39	28	17	59	51	46
RCL	0.944 ± 0.092	0.905 ± 0.101	0.891 ± 0.100	0.891 ± 0.101	0.858 ± 0.098	0.807 ± 0.075	0.794 ± 0.041	0.749 ± 0.032	0.780 ± 0.018	**0.894 ± 0.109**	0.819 ± 0.089	0.817 ± 0.090
PRC	0.820 ± 0.083	0.793 ± 0.085	0.770 ± 0.085	0.77 ± 0.087	0.721 ± 0.092	0.640 ± 0.086	0.587 ± 0.063	0.569 ± 0.062	0.596 ± 0.069	**0.893 ± 0.044**	0.828 ± 0.067	0.828 ± 0.067
F1-score	0.871 ± 0.053	0.838 ± 0.061	0.818 ± 0.06	0.818 ± 0.060	0.775 ± 0.059	0.707 ± 0.051	0.672 ± 0.038	0.644 ± 0.042	0.673 ± 0.042	**0.89 ± 0.069**	0.82 ± 0.066	0.819 ± 0.066
AUC	0.936 ± 0.038	0.885 ± 0.050	0.863 ± 0.055	0.863 ± 0.054	0.806 ± 0.070	0.682 ± 0.115	0.576 ± 0.14	0.551 ± 0.149	0.614 ± 0.112	**0.940 ± 0.052**	0.901 ± 0.054	0.900 ± 0.054
ACC	0.86 ± 0.061	0.824 ± 0.069	0.802 ± 0.071	0.801 ± 0.071	0.749 ± 0.078	0.661 ± 0.086	0.608 ± 0.074	0.581 ± 0.079	0.616 ± 0.074	**0.893 ± 0.058**	0.821 ± 0.062	0.821 ± 0.062
BACC	0.86 ± 0.061	0.824 ± 0.069	0.802 ± 0.071	0.801 ± 0.071	0.749 ± 0.078	0.661 ± 0.086	0.608 ± 0.074	0.581 ± 0.079	0.616 ± 0.074	**0.893 ± 0.058**	0.821 ± 0.062	0.821 ± 0.062

### Risk factors of ELOHS

Table 5 shows the risk factors and relative risk (RR) of ELOHS determined at a 95% significant level. The reference sub-features for the multivariate LR are ADC (PL1), ADT (M), CCI (0), DTH (0-5 km), PAG (20–50 years), PGD (female), PRG (no religion), VMO (orthopaedic surgery), and SES (high). As expected, PAG is the predominant risk factor for ELOHS with PAG (> 90) {RR: 1.85 (1.34–2.56), *P*:  < 0.001} having 6.32% more likelihood of ELOHS compared with PAG (80–90) {RR: 1.74 (1.34–2.38), *P*:  < 0.001} and 23.3% more susceptible than PAG (70–80) {RR: 1.5 (1.1–2.05), *P*: 0.011}. Patients who are from ADC (US1) {RR: 3.64 (3.09–4.28, *P*:  < 0.001} are 14.8% and 70.5%, respectively, more prone to ELOHS compared to ADC (UC1) {RR: 3.17 (2.82–3.55), *P*:  < 0.001} and ADC (EMG) {RR: 2.11 (1.93–2.31), *P*:  < 0.001}. However, patients who fell into the ADC (others) {RR: 4.11 (2.71–6.24), *P*:  < 0.001} are 12.9% and 94.8%, respectively, more likely to have ELOHS compared to those admitted under the ADC (US1) and ADC (EMG) categories. Patients from SES (Low) {RR: 1.45 (1.24–1.71), *P*:  < 0.001)} are 13.3% more likely to have ELOHS compared to those from SES (middle) {RR: 1.28 (1.1–1.5), *P*: 0.002} and 45% more likely than SES (high) used as the reference for the SES categories. The remaining risk factors such as CCI, DTH and some VMO specialties such as breast surgery, cardiology, endocrine surgery, etc., have a limited likelihood of influencing ELOHS since their RR are < 1.

**Table 5:** Prediction accuracy of ELOHS and NLOHS with RFECV-ETC algorithm for the best model (T#10)

Folds	Class	Precision	Recall	F1 Score	BACC	AUC
1	NLOHS	0.7	0.91	0.79	0.7612	0.8135
	ELOHS	0.87	0.61	0.72		
2	NLOHS	0.79	0.89	0.84	0.8297	0.8707
	ELOHS	0.88	0.77	0.82		
3	NLOHS	0.95	0.94	0.94	0.9442	0.9761
	ELOHS	0.94	0.95	0.94		
4	NLOHS	0.94	0.89	0.91	0.913	0.9619
	ELOHS	0.89	0.94	0.92		
5	NLOHS	0.94	0.95	0.95	0.9452	0.9807
	ELOHS	0.95	0.94	0.94		
6	NLOHS	0.94	0.9	0.92	0.9216	0.962
	ELOHS	0.91	0.94	0.92		
7	NLOHS	0.94	0.91	0.93	0.928	0.9712
	ELOHS	0.91	0.95	0.93		
8	NLOHS	0.94	0.73	0.82	0.8443	0.9375
	ELOHS	0.78	0.95	0.86		
9	NLOHS	0.94	0.91	0.93	0.9271	0.9722
	ELOHS	0.91	0.94	0.93		
10	NLOHS	0.94	0.87	0.91	0.9108	0.9562
	ELOHS	0.88	0.95	0.91		

**Fig. 3:**
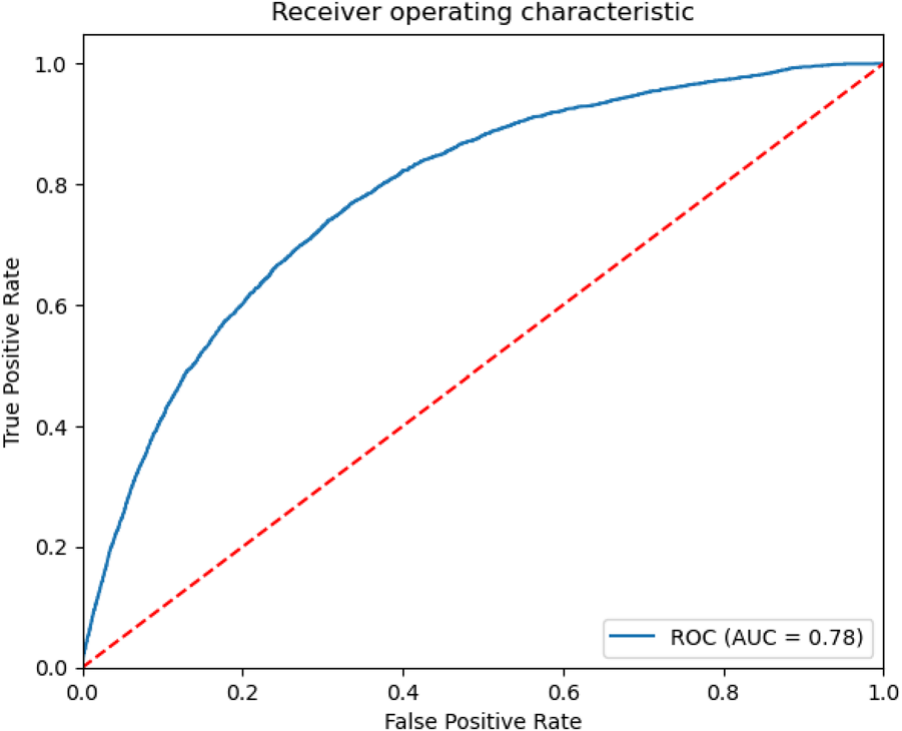
Receiver operating characteristic (ROC) area under the curve (AUC) for the multivariate logistic regression used for determining the risk factors and relative risks

The accuracy of the multivariate LR used for estimating the risk factors and RR of the features is computed with ROC AUC shown in Fig. 3.

Following the information in Table 5, the severity of the risk factors of ELOHS is grouped as patient features, DRG specialty and hospital-based features in Table 6. For the patients’ features, PAG (> 90) is the most profound risks factor with 6.32—2983% more likelihood of contributing to ELOHS compared to the other risk factors shown in Table 7. The risk factors associated with ADC (US1, others, UC1, EMG) have higher risk severities than the rest of the hospital-based features contributing to ELOHS. The risk of ELOHS associated with the various DRGs are comparatively lower than those associated with patients and hospital-based features and may have less likelihood of triggering ELOHS for patients treated for different health conditions.

**Table 6:** Summary of the risk factors of ELOHS for all patients showing the relative risks (RR) and P-values obtained from a multivariate logistic model (* are significant features at 95% level)

Parameters	Size	RR (95% CI)	P-value	Parameters	size	RR (95% CI)	P-value
VMO specialty				Admission type			
Orthopaedic surgery	3379	Ref.		M	11,943	Ref.	
Breast surgery	380	0.19 (0.14–0.28)	< 0.001*	AS	3892	0.84 (0.73–0.95)	0.008*
Cardiology	3313	0.25 (0.22–0.28)	< 0.001*	AS2	2494	1.99 (1.73–2.29)	< 0.001*
Cardiothoracic Surg	701	0.33 (0.25–0.44)	< 0.001*	AS3	1015	0.95 (0.78–1.17)	0.634
Colorectal surgery	1674	0.28 (0.24–0.34)	< 0.001*	AS4	808	0.46 (0.36–0.6)	< 0.001*
ENT surgery	1175	0.12 (0.09–0.17)	< 0.001*	CA	173	0.16 (0.06–0.46)	< 0.001*
Emergency physician	321	n/a	0.999	M2	393	1.86 (1.48–2.34)	< 0.001*
Endocrine surgery	219	0.09 (0.04–0.17)	< 0.001*	M3	604	0.84 (0.67–1.03)	0.097
Endocrinology	620	0.41 (0.33–0.5)	< 0.001*	NEW	140	2.77 (1.24–6.17)	0.013*
Gastroenterology	1630	0.28 (0.23–0.32)	< 0.001*	O3	111	n/a	0.999
General Medicine Phy	2349	0.3 (0.26–0.35)	< 0.001*	OBC	724	0.28 (0.11–0.7)	0.007*
General Paed. Surg	112	0.94 (0.38–2.32)	0.886	OBN	1039	0.03 (0.01–0.14)	< 0.001*
General Paed.Med	340	0.33 (0.17–0.67)	0.002*	Others	558	1.81 (1.45–2.26)	< 0.001*
Gerontology	1285	0.28 (0.24–0.34)	< 0.001*	S	8520	1.37 (1.25–1.5)	< 0.001*
Gynaecology	464	0.17 (0.12–0.24)	< 0.001*	S2	2214	1.71 (1.5–1.96)	< 0.001*
Haematology	619	0.34 (0.27–0.43)	< 0.001*	Charlson Score			
Hepato/biliary/pancr	693	0.17 (0.12–0.22)	< 0.001*	0	7994	Ref	< 0.001*
Infectious disease	184	0.69 (0.49–0.98)	0.035*	1	3020	0.34 (0.25–0.47)	< 0.001*
Medical oncology	1374	0.25 (0.21–0.31)	< 0.001*	2	4230	0.32 (0.23–0.43)	< 0.001*
Nephrology	839	0.54 (0.45–0.65)	< 0.001*	3	5751	0.31 (0.22–0.42)	< 0.001*
Neurology	824	0.46 (0.38–0.55)	< 0.001*	4	7658	0.39 (0.29–0.54)	< 0.001*
Neurosurgery	1204	0.17 (0.13–0.22)	< 0.001*	5	2844	0.43 (0.31–0.6)	< 0.001*
Obstetrics	109	n/a	0.999	6	1357	0.52 (0.37–0.72)	< 0.001*
Obstetrics & Gynae	2063	0.06 (0.04–0.1)	< 0.001*	7	546	0.68 (0.47–0.99)	0.043*
Ophthalmic surgery	302	0.08 (0.04–0.15)	< 0.001*	> 8	1228	0.56 (0.4–0.79)	0.001*
Plastic/recon surg	1822	0.44 (0.38–0.51)	< 0.001*	Patient religion			
Respiratory medicine	1054	0.28 (0.23–0.33)	< 0.001	No religion	8441	Ref	
Trainee	282	n/a	0.999	Anglican	4714	0.78 (0.7–0.87)	< 0.001*
Upper GI surgery	1358	0.15 (0.12–0.18)	< 0.001*	Baptist	170	1.04 (0.68–1.6)	0.84
Urogynaecology	134	0.08 (0.03–0.19)	< 0.001*	Catholic	7224	0.77 (0.7–0.84)	< 0.001*
Urology	2493	0.25 (0.21–0.29)	< 0.001*	Christian	1396	0.51 (0.42–0.62)	< 0.001*
Vascular surgery	711	0.38 (0.3–0.47)	< 0.001*	Christian (others)	392	0.59 (0.42–0.82)	0.002*
Others	601	0.43 (0.34–0.55)	< 0.001*	Greek Orthodox	1109	0.62 (0.51–0.75)	< 0.001*
Patient age (years)				Jewish	3513	0.62 (0.55–0.7)	< 0.001*
20–50	Ref.			Lutheran	135	0.62 (0.37–1.04)	0.069
50–60	3386	0.99 (0.73–1.34)	0.95	Methodist	112	0.68 (0.4–1.16)	0.161
60–70	5202	1.19 (0.87–1.62)	0.272	Presbyterian	571	0.8 (0.64–1.02)	0.067
70–80	7409	1.5 (1.1–2.05)	0.011*	Protestant	302	0.99 (0.73–1.33)	0.935
80–90	6764	1.74 (1.27–2.38)	< 0.001*	Religion (others)	894	0.53 (0.41–0.68)	< 0.001*
< 20	1794	0.06 (0.04–0.09)	< 0.001*	Undefined	4444	0.53 (0.47–0.6)	< 0.001*
> 90	3524	1.85 (1.34–2.56)	< 0.001*	Uniting church	1211	0.81 (0.68–0.96)	0.013*
Patient gender				Distance to hospital (km)			
Female	19,887	Ref.		0-5 km	11,519	Ref.	
Male	14,741	0.63 (0.59–0.67)	< 0.001*	> 20 km	7284	0.64 (0.56–0.73)	< 0.001*
Admission category				5-10 km	8936	0.72 (0.67–0.78)	< 0.001*
PL1	16,734	Ref.	< 0.001*	10-20 km	6889	0.75 (0.68–0.82)	< 0.001*
EMG	11,619	2.11 (1.93–2.31)	< 0.001*	Socioeconomic status			
MAT	1865	1.41 (0.64–3.11)	0.39	High	29,131	Ref.	
Others	258	4.11 (2.71–6.24)	< 0.001*	Middle	2998	1.28 (1.1–1.5)	0.002*
UC1	2739	3.17 (2.82–3.55)	< 0.001*	Low	2487	1.45 (1.24–1.7)	< 0.001*
US1	1413	3.64 (3.09–4.28)	< 0.001*

**Table 7:** Risk severity of the various risk factors of ELOHS (NB: all features are computed at 95% significance level; ** are significant at 90% significance level)

Parameter	RR (95%CI)	Parameter	RR (95%CI)
*Patients' features*		*DRG specialty*	
PAG (> 90)	1.85 (1.34–2.56)	VMO (infectious disease)	0.69 (0.49–0.98)
PAG (80–90)	1.74 (1.27–2.38)	VMO (nephrology)	0.54 (0.45–0.65)
PAG (70–80)	1.5 (1.1–2.05)	VMO (neurology)	0.46 (0.38–0.55)
SES (low)	1.45 (1.24–1.7)	VMO (plastic/recon surg)	0.44 (0.38–0.51)
SES (middle)	1.28 (1.1–1.5)	VMO (others)	0.43 (0.34–0.55)
PRG (uniting church)	0.81 (0.68–0.96)	VMO (endocrinology)	0.41 (0.33–0.5)
PRG (Anglican)	0.78 (0.7–0.87)	VMO (vascular surgery)	0.38 (0.3–0.47)
PRG (Catholic)	0.77 (0.7–0.84)	VMO (haematology)	0.34 (0.27–0.43)
DTH (10-20 km)	0.75 (0.68–0.82)	VMO (general paed.med.)	0.33 (0.17–0.67)
DTH (5-10 km)	0.72 (0.67–0.78)	VMO (cardiothoracic surg.)	0.33 (0.25–0.44)
CCI (7)	0.68 (0.47–0.99)	VMO (general medicine phy)	0.3 (0.26–0.35)
DTH (> 20 km)	0.64 (0.56–0.73)	VMO (colorectal surgery)	0.28 (0.24–0.34)
PGD (male)	0.63 (0.59–0.67)	VMO (gerontology)	0.28 (0.24–0.34)
PRG (Jewish)	0.62 (0.55–0.7)	VMO (respiratory medicine)	0.28 (0.23–0.33)
PRG (Greek Orthodox)	0.62 (0.51–0.75)	VMO (gastroenterology)	0.28 (0.23–0.32)
PRG (Christian (others))	0.59 (0.42–0.82)	VMO (medical oncology)	0.25 (0.21–0.31)
CCI (> 8)	0.56 (0.4–0.79)	VMO (urology)	0.25 (0.21–0.29)
PRG (undefined)	0.53 (0.47–0.6)	VMO (cardiology)	0.25 (0.22–0.28)
PRG (religion (others))	0.53 (0.41–0.68)	VMO (breast surgery)	0.19 (0.14–0.28)
CCI (6)	0.52 (0.37–0.72)	VMO (gynaecology)	0.17 (0.12–0.24)
PRG (Christian)	0.51 (0.42–0.62)	VMO (neurosurgery)	0.17 (0.13–0.22)
CCI (5)	0.43 (0.31–0.6)	VMO (hepato/biliary/pancr)	0.17 (0.12–0.22)
CCI (4)	0.39 (0.29–0.54)	VMO (upper GI surgery)	0.15 (0.12–0.18)
CCI (1)	0.34 (0.25–0.47)	VMO (ENT surgery)	0.12 (0.09–0.17)
CCI (2)	0.32 (0.23–0.43)	VMO (endocrine surgery)	0.09 (0.04–0.17)
CCI (3)	0.31 (0.22–0.42)	VMO (urogynaecology)	0.08 (0.03–0.19)
PAG (< 20)	0.06 (0.04–0.09)	VMO (ophthalmic surgery)	0.08 (0.04–0.15)
		VMO (obstetrics & gynae)	0.06 (0.04–0.1)
*Hospital-based features*		*DRG specialty*	
ADC (others)	4.11 (2.71–6.24)	ADT (others)	1.81 (1.45–2.26)
ADC (US1)	3.64 (3.09–4.28)	ADT (S2)	1.71 (1.5–1.96)
ADC (UC1)	3.17 (2.82–3.55)	ADT (S)	1.37 (1.25–1.5)
ADT (NEW)	2.77 (1.24–6.17)	ADT (AS)	0.84 (0.73–0.95)
ADC (EMG)	2.11 (1.93–2.31)	ADT (AS4)	0.46 (0.36–0.6)
ADT (AS2)	1.99 (1.73–2.29)	ADT (OBC)	0.28 (0.11–0.7)
ADT (M2)	1.86 (1.48–2.34)	ADT (CA)	0.16 (0.06–0.46)
		ADT (OBN)	0.03 (0.01–0.14)

## Discussion

This study develops a preadmission assessment for patients admitted to a private acute teaching hospital to predict those that are prone to ELOHS and identify the risk factors of ELOHS to enable hospitals to pro-actively plan their care. By relying on hospital-specific features and patients' demographic and psychosocial characteristics, it was possible to develop a machine learning algorithm for reasonably identifying patients that will exceed their expected length of stay on admission. It can be inferred from the study that the risk of ELOHS is very predominant for patients who are 60 years and over and those that have been treated for infectious disease and neurological conditions. Even though some other conditions considered in the study such as neurosurgery, upper GI surgery, urology, cardiology, etc. (see VMO specialty in Table 5) are also linked to ELOHS because they are significant at 95% confidence level, the fact that the RR of these factors are small (< 1) means that their tendency of causing ELOHS is minimal compared to those with the RR values > 1.

The influence of age on ELOHS is pronounced as the rate of ELOHS increases with the age of the patients, a finding that resonated with other researchers, who also attributed SES, which is a risk factor in this study to ELOHS [6–9]. The risk of ELOHS is pronounced with patients who are ≥ 80 years judging from their RR of > 1, but previous research showed that those who are prone to ELOHS are patients who are ≥ 65 years old for patients of elective anterior cervical discectomy and fusion [7]. It is important to note that the vulnerability of the elderly can be attributed to hospital-acquired infections and other complications in hospitals [17]. This and other factors combine to cause complications, which result in a higher likelihood of ELOHS amongst the elderly [6]. This situation has resulted in higher hospitals costs, shortage of hospital space, and poor-quality penalties imposed on hospitals [18, 19]. There are significant incentives for hospitals to improve patients’ outcomes through quality care that will reduce hospital-acquired infections from endogenous and procedure-related risk factors [10, 11].

Previous studies linked Charlson score, which can be a good gauge of a patient’s comorbidity predisposition [12] to ELOHS. For this study, Charlson scores of 1–6 are risk factors of ELOHS, however, since the relative risk (RR: 0.04–0.09; *P*-value:  < 0.001)) of the patients is < 1, there is a higher likelihood they may not exceed their expected stay on admission. Despite the impact of Charlson score on ELOHS, it is also linked to unplanned readmission due to the severity of comorbidities [13]. This also suggests that the current DRG models successfully account for patient complexity.

The prediction accuracy of the ELOHS model, which is 89.3% is comparatively higher than the accuracy obtained by other researchers [9, 16, 20, 35] even though it may be difficult to justify some of their techniques for defining ELOHS. This is because some of the patients who may have been classified as likely to exceed their expected length of stay in the hospital because they spent 3, 4, 5, 9, or 11 days based on the proposition of the models may have not exceed their expected length of stay in the hospital following the assessment of their DRG per the technique described in this study. Even though most of the studies reported on specific disease conditions [9, 21, 35], the current study painted a better picture of ELOHS by taking a comprehensive look at patients in the acute care setting. This approach gives the hospital a better tool for an immediate decision on requisite patients’ management plans to forestall complications that will result in ELOHS. Again, it is important to state that some of the ELOHS contributing features investigated by many previous researchers such as surgical approach, preoperative functional status, and patients’ anaesthetic history [36, 37] may not suffice for preadmission assessment of ELOHS.

The core limitation of this study is the few features considered. There is a need to consider more demographic and psychosocial features such as ethnicity, level of education, marital status, and the comorbidities suffered by these patients as they have the potentials of influencing ELOHS. There is also the need to increase the data size while looking at a narrowed classification of similar DRGs to facilitate better accuracy of the prediction model. Again, the reliance on only 10 hospital-specific and psychosocial features for the analysis may not suffice. Other important features that can contribute to ELOHS such as the kind of procedure adopted for surgical patients will be vital for consideration in future studies.

## Conclusions

To ensure that patients who are prone to ELOHS are given appropriate, tailored care when admitted to the hospital, a technique for preadmission assessment with hospital-specific and psychosocial features is developed in this study using hospital records. By relying on RFECV-ETC algorithm that uses the backward elimination technique, and ETC as the base estimator, it was possible to develop a model that predicted patients expected to have ELOHS. The study relied on SMOTE up sampling, tenfold cross-validation, and features such as VMO specialty, patient age, patient gender, admission category, admission type, patient care class, patient religion, distance to hospital, SES, and Charlson score. After 12 trials of different combinations of the features, the model with the best accuracy predicted ELOHS to 89.3% accuracy, 89.4% recall, 89.4% precision, and identified 61 optimal sub-features for ELOHS prediction.

Since the knowledge of the risk factors of ELOHS is vital for developing strategies for better care outcomes, multivariate LR was used for estimating the risk factors of ELOHS at a 95% confidence level and the relative risk of the risk factors. The risk of ELOHS increases with age, ADC (EMG, UC1, US1 and others), ADT (M2, AS2, NEW, S, S2, others), SES (low, middle), etc., while the VMO specialties have limited likelihood of increasing ELOHS despite many of them being risk factors of ELOHS. Prediction of ELOHS before admitting the patients and understanding the risk factors of ELOHS will make patient management better because of the increased likelihood of implementing adequate and person-centred treatments.

## Summary points

### What is known

- Extended length of hospital stay has been treated as a LOS more than a given number of days of hospitalization such as 4, 5, 6, 9, etc.
- Charlson scores affect LOS in hospitalization.
- ELOHS is computed for patients already admitted to the hospital using clinical characteristics.
- ELOHS has been treated for specific disease conditions, not in consideration of different DRGs.

### What was found

- Extended length of hospital stay (ELOHS) was determined as 3* average LOS for a given DRG.
- ELOHS can be predicted preadmission to 89.3% accuracy with RFECV-ETC.
- Infectious diseases and neurology patients have a very high likelihood of ELOHS compared to patients admitted for other DRGs.
- The rate of ELOHS amongst patients increases with age but patients who are ≥ 80 years have a higher likelihood of ELOHS than younger patients.
- Charlson score is a risk factor of ELOHS, but has a limited likelihood of causing ELOHS due to the low relative risk (< 1.0).

## Data Availability

Can be made available on request.

## Appendix I

**Table:**

Characteristics of algorithms	
Algorithm	Characteristics
KNN	KNeighborsClassifier (algorithm = 'auto', leaf_size = 30, metric = 'minkowski', metric_params = None, n_jobs = None, n_neighbors = 5, p = 2, weights = 'uniform')
GBM	Gradient Boosting Classifier (ccp_alpha = 0.0, criterion = 'friedman_mse', init = None, learning_rate = 0.1, loss = 'deviance', max_depth = 3, max_features = None, leaf_nodes = None, min_impurity_decrease = 0.0, min_impurity_split = None, min_samples_leaf = 1, min_samples_split = 2, min_weight_fraction_leaf = 0.0, n_estimators = 100, n_iter_no_change = None, presort = 'deprecated', random_state = None, subsample = 1.0, tol = 0.0001, validation_fraction = 0.1, verbose = 0, warm_start = False)
ADB	Ada Boost Classifier (algorithm = 'SAMME.R', base_estimator = None, learning_rate = 1.0, n_estimators = 50, random_state = None)
ETC	Extra Trees Classifier (bootstrap = False, ccp_alpha = 0.0, class_weight = None, criterion = 'gini', max_depth = None, max_features = 'auto', max_leaf_nodes = None, max_samples = None, min_impurity_decrease = 0.0, min_impurity_split = None,min_samples_leaf = 1, min_samples_split = 2, min_weight_fraction_leaf = 0.0, n_estimators = 100, n_jobs = None, oob_score = False, random_state = None, verbose = 0, warm_start = False)
SVM	SVC (C = 1.0, break_ties = False, cache_size = 200, class_weight = None, coef0 = 0.0, decision_function_shape = 'ovr', degree = 3, gamma = 'scale', kernel = 'rbf', max_iter = -1, probability = False, random_state = None, shrinking = True, tol = 0.001, verbose = False)
XGB	XGBClassifier (base_score = 0.5, booster = 'gbtree', colsample_bylevel = 1, colsample_bynode = 1, colsample_bytree = 1, gamma = 0, gpu_id = -1, importance_type = 'gain', interaction_constraints = '', learning_rate = 0.300000012, max_delta_step = 0, max_depth = 6, min_child_weight = 1, missing = nan, monotone_constraints = ' ()', n_estimators = 100, n_jobs = 4, num_parallel_tree = 1, objective = 'binary: logistic', random_state = 0, reg_alpha = 0, reg_lambda = 1, scale_pos_weight = 1, subsample = 1, tree_method = 'exact', use_label_encoder = True, validate_parameters = 1, verbosity = None)
RF	Random Forest Classifier (bootstrap = True, ccp_alpha = 0.0, class_weight = None, criterion = 'gini', max_depth = None, max_features = 'auto', max_leaf_nodes = None, max_samples = None, min_impurity_decrease = 0.0, min_impurity_split = None, min_samples_leaf = 1, min_samples_split = 2, min_weight_fraction_leaf = 0.0, n_estimators = 100, n_jobs = None, oob_score = False, random_state = None, verbose = 0, warm_start = False)

## Abbreviations

ACC: Accuracy
ADB: Adaptive booster
ADC: Admission category
PL1: Planned
EMG: Emergency (via ED)
MAT: Maternity
UC1: Unscheduled community
US1: Unscheduled (usually from the specialist’s rooms)
Others: Includes CA1 (admissions medical (PAL, Rehab), NEW (newborns), STA/STD (statistical admissions/discharges)
ADT: Admission type
M: Medical
M2: Medical 2
M3: Medical 3
S: Surgical
S2: Surgical 2
AS: Advanced surgical
AS2: Advanced surgical 2
AS3: Advanced surgical 3
AS4: Advanced surgical 4
CA: Normally within the admission category group
NEW: Newborn
O3: Overnight Band 3 (usually used for endoscopy patients)
OBC: Obstetrics deliveries meant to be used for C section
OBN: Obstetrics meant to be used for vaginal deliveries
Others: Number codes, HITH, day procedures, ON exception procedures,
ALOS: Average length of stay
ANN: Artificial Neural Network
AUC: Area under the curve
BACC: Balanced accuracy
CCI: Charlson Score
CI: Confidence interval
COPD: Chronic obstructive pulmonary disease
DRG: Diagnosis related group
DTC: Decision tree classifier
DTH: Distance to hospital
ELOHS: Extended length of stay
ETC: Extra tree classifier
GBM: Gradient boosting machine
GPS: Global Positioning System
ICU: Intensive care unit
LOS: Length of stay
LR: Logistic regression
NLOHS: Normal length of hospital stay
OFS: Optimal features selection
PAG: Patient age
PCC: Patient care class
UNQ: Unqualified new born
REH: Rehabilitation
ACU: Acute
PAL: Palliative care
PGD: Patient gender
PRC: Precision
PRG: Patient religion
RCL: Recall
RF: Random Forest
RFECV-ETC: Recursive Feature Elimination with Cross-Validation and Extra Tree Classifier
RFECV: Recursive Feature Elimination with Cross-Validation
RR: Relative risk
SEIFA: Socio-economic indexes for areas
SES: Socioeconomic status
SMOTE: Synthetic minority oversampling technique
SVM: Support vector machine
VMO: Visiting medical officer
XGB: XGBoost
